# Transient presence of stomatocytes: A clue to the diagnosis of overhydrated hereditary stomatocytosis in a child with beta‐thalassemia

**DOI:** 10.1002/jcla.24991

**Published:** 2023-12-13

**Authors:** Yanhui Chen, Qile Lin, Wenpeng Ni, Kunyi Deng, Lilian Li

**Affiliations:** ^1^ Clinical Laboratory Zhongshan Boai Hospital Affiliated to Southern Medical University Zhongshan Guangdong China; ^2^ Clinical Laboratory Zhongshan Guzhen People's Hospital Zhongshan Guangdong China

**Keywords:** hemolytic anemia, hereditary overhydrated stomatocytosis, stomatocytes

## Abstract

**Background:**

Overhydrated hereditary stomatocytosis (OHSt) is a rare disorder characterized by abnormalities in erythrocytic volume homeostasis. Early and accurate diagnosis is essential for appropriate management and genetic counseling.

**Methods:**

We present the case of a child with beta‐thalassemia and a history of multiple blood transfusions. Clinical presentation, laboratory findings, and genetic testing were reviewed. Peripheral blood smear examination and genetic analysis were performed.

**Results:**

The patient was admitted with severe anemia, and peripheral blood smear examination revealed the presence of up to 50% stomatocytes. Laboratory investigations showed abnormalities in red blood cell parameters, including decreased hemoglobin levels and increased mean corpuscular volume. Genetic testing identified a heterozygous mutation in the *RHAG* gene, confirming the diagnosis of OHSt. The presence of stomatocytes in the peripheral blood smear was transient, correlating with episodes of hemolysis and its control.

## 1 INTRODUCTION

Hereditary stomatocytosis is a rare disorder characterized by an abnormality in erythrocytic volume homeostasis.^1^ It is classified into three main types based on the concentration of Na^+^ and K^+^ in stomatocytes: the dehydrated type, also known as hereditary dehydrated stomatocytosis (DHSt); the overhydrated type, also known as OHSt; and the intermediate syndrome.^2^ OHSt is an autosomal dominant condition caused by heterozygous mutations in genes encoding membrane transporters such as *RHAG*, *SLC4A1*, and *SLC2A1*.^3^, ^4^, ^5^

In OHSt, there is an increased permeability of the erythrocyte membrane to monovalent cations, leading to elevated intracellular Na^+^ concentration, reduced K^+^ concentration, and subsequent water influx into the cells.^6^ These cellular changes ultimately result in increased osmotic fragility of erythrocytes and their premature destruction within the spleen.^7^ Clinically, patients with OHSt often present with varying degrees of anemia and reticulocytosis, along with the presence of red blood cells exhibiting slit‐like stomatocytic lucencies on blood smears. However, due to the rarity of this disease and its variable phenotype, accurate diagnosis can be challenging. Additionally, stomatocytes may be overlooked or dismissed as artifacts in routine clinical laboratory evaluations.

Here, we present a case of a child who was repeatedly hospitalized with fever and anemia, and was eventually diagnosed with OHSt. This case highlights the importance of considering hereditary stomatocytosis, particularly OHSt, as a differential diagnosis in patients with unexplained anemia and characteristic red blood cell morphology. Early recognition and accurate diagnosis are crucial for appropriate management and genetic counseling in affected individuals.

## 2 CASE PRESENTATION

**FIGURE 1:**
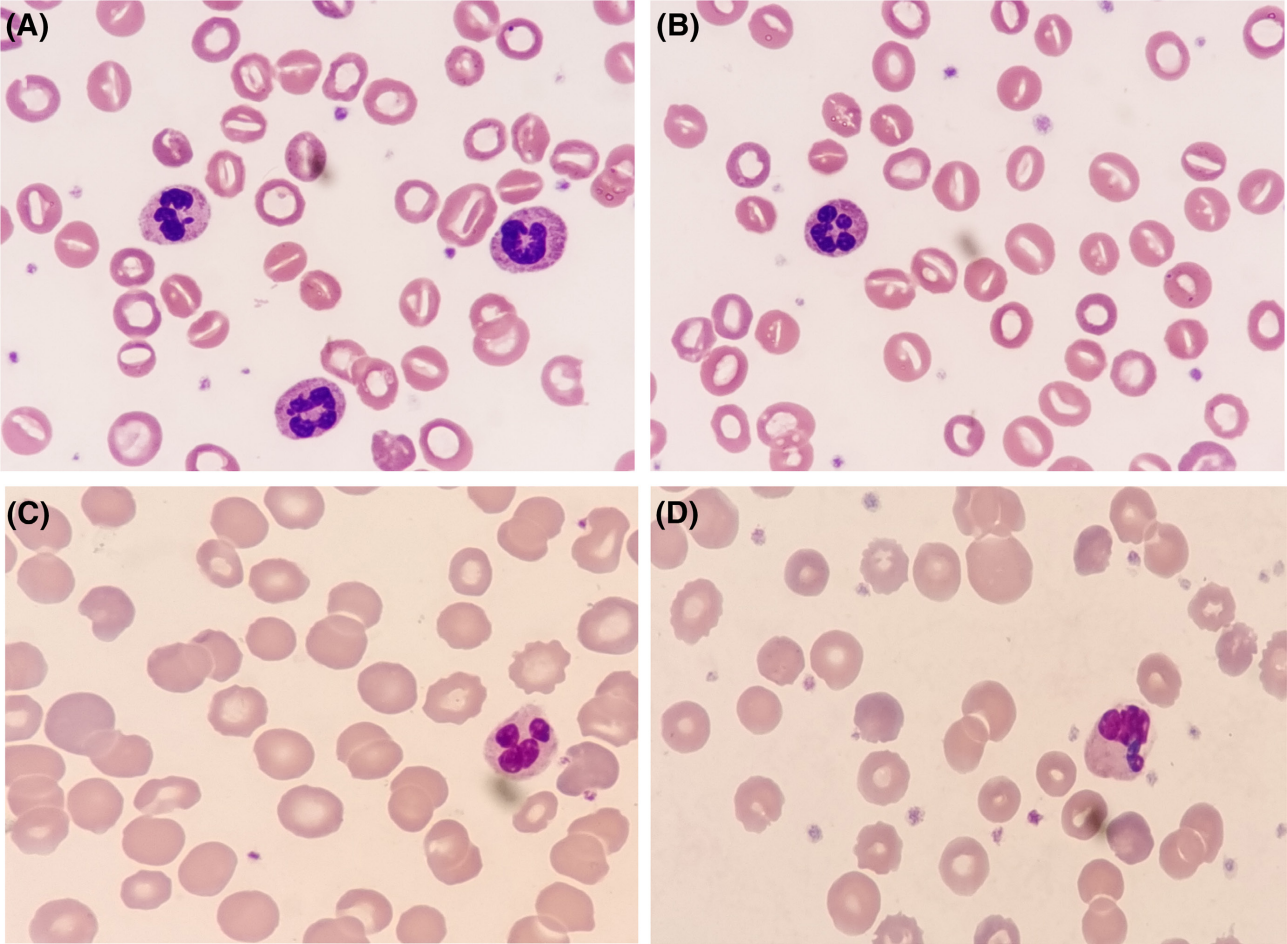
Cytomorphological examination of peripheral blood. (A) and (B) A large number of stomatocytes can be seen in the blood smear; (C) morphological examination of the same blood sample 24 h later shows no stomatocytes; (D) no stomatocytes are found in the patient's peripheral blood 18 h after blood transfusion, Wright staining, ×1000.

A female patient with beta‐thalassemia (*Cap/N*) had a history of receiving 18 blood transfusions for severe anemia during the first 9 years of her life. No other abnormalities were detected by physicians during this period. However, the patient's condition took a surprising turn when she was readmitted to the hospital in January 2022. Laboratory tests revealed several abnormal findings. The leukocyte count was elevated at 22.92 (reference range: 4.3–14.2) × 109/L, the erythrocyte count was decreased to 1.52 (reference range: 4.2–5.7) × 1012/L, and the hemoglobin level was severely reduced to 55 (reference range: 118–156) g/L. Mean corpuscular volume (MCV) was elevated at 140.2 (reference range: 77–92) fl, and mean corpuscular hemoglobin concentration (MCHC) was decreased to 229 (reference range: 310–355) g/L. The reticulocyte count was elevated at 0.57 (reference range: 0.02–0.20) × 1012/L, indicating an increased rate of red blood cell production. The patient's total bilirubin was significantly elevated at 128.9 (reference range: 1.71–17.1) μmoL/L, with direct bilirubin at 18.5 (reference range: 1.71–7.0) μmoL/L and indirect bilirubin at 110.4 (reference range: 1.7–13.7) μmol/L. Ferritin levels were also elevated at 1926.0 (reference range: 18–91) ng/mL. The direct antiglobulin test was negative, indicating the absence of immune‐mediated hemolysis. Increased red blood cell fragility and a plasma free hemoglobin level of 30 (<4) mg/dL further supported the diagnosis of hemolysis. Notably, up to 50% of stomatocytes were observed in the peripheral blood smear (Figure 1A,B).

**FIGURE 2:**
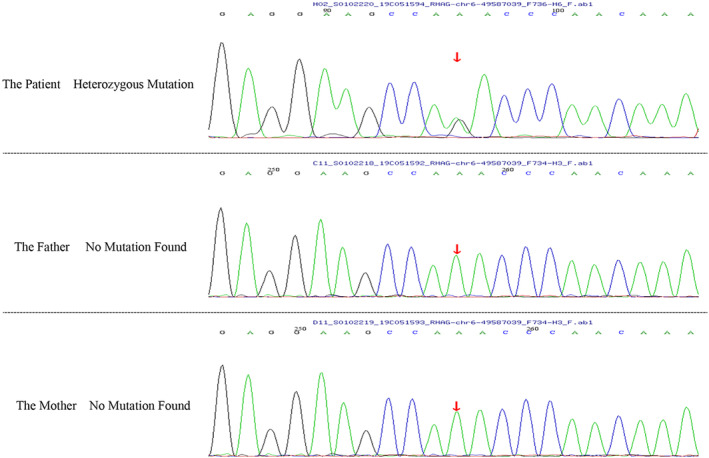
Sequencing results show that the patient has a heterozygous mutation in the RHAG gene and no abnormality in the parents.

Subsequent high‐throughput sequencing identified a heterozygous mutation in the *RHAG* gene (chr6:4958 7039; NM_0003 24; exon2; c.194 T > C [p.F65S]) (Figure 2). Pedigree analysis did not reveal the presence of this mutation in the patient's parents, suggesting a spontaneous occurrence. The patient was ultimately diagnosed with hereditary overhydrated stomatocytosis (OHSt).

However, a puzzling aspect of this case was the absence of stomatocytes in the patient's blood samples during multiple morphological examinations over the past 9 years. Surprisingly, when a blood smear was performed again on the same sample after 24 h, all the stomatocytes had disappeared (Figure 1C). Additionally, nearly normal red blood cells were observed in the patient's peripheral blood 18 h after receiving a blood transfusion (Figure 1D). Considering the patient's regular blood transfusions and the timing of this admission, it is speculated that the large number of stomatocytes observed in the blood smear was due to timely blood sample collection and the limited effect of the blood transfusion.

## 3 DISCUSSION

OHSt, first reported by Miller et al. in the late 1960s to early 1970s,^8^, ^9^ remains a rare disorder with limited treatment options available. The pathogenesis of OHSt involves mutations in genes encoding membrane transporters, leading to increased erythrocyte membrane permeability to monovalent cations, altered hydration status and subsequent stomatocyte formation. Despite advancements in our understanding of the disease, there is still no definitive cure for OHSt, and treatment mainly focuses on managing symptoms and complications.

Splenectomy has been reported as a potential treatment option for OHSt, with varying outcomes reported in the literature.^10^ Therefore, the decision to perform splenectomy in OHSt should be individualized, considering the patient's clinical presentation, severity of symptoms and potential risks associated with the procedure.

The diagnosis of OHSt can be challenging, particularly when the number of stomatocytes in the blood smear is small or when their morphology is atypical. Additionally, the presence of other factors that can interfere with red blood cell morphology can further complicate the diagnosis. However, despite these challenges, OHSt should be thoroughly considered in the differential diagnosis of hemolytic anemia, including in newborns. The combination of clinical findings, peripheral blood cell morphology, and genetic diagnosis can provide valuable insights for the accurate and timely diagnosis of OHSt.

In the present case, the observation of stomatocytes in the patient's peripheral blood smear during episodes of hemolysis and their subsequent disappearance when hemolysis was controlled is notable. This finding suggests that the presence of stomatocytes in the peripheral blood is closely related to the degree of hemolysis and hydration status of the erythrocytes. These dynamic changes in stomatocyte morphology emphasize the importance of timely specimen acquisition and careful interpretation of blood smear findings in suspected cases of OHSt.

Early diagnosis of OHSt is essential for appropriate management and genetic counseling. Although there is currently no definitive cure for OHSt, supportive measures such as regular blood transfusions and iron supplementation can help manage anemia and alleviate symptoms. Additionally, close monitoring of the patient's clinical and laboratory parameters is necessary to assess treatment response and identify any potential complications associated with chronic anemia.

In conclusion, OHSt is a rare disorder that can present with variable clinical features and challenges in diagnosis. The dynamic nature of stomatocyte presence in peripheral blood smears underscores the significance of timely specimen collection and careful evaluation of medical history in identifying and diagnosing OHSt. Further research and clinical studies are warranted to enhance our understanding of this rare condition and explore potential therapeutic interventions.

## AUTHOR CONTRIBUTIONS

Wenpeng Ni, Yanhui Chen, and Lilian Li collected and analyzed data. Wenpeng Ni and Qile Lin wrote the manuscript. Kunyi Deng revised the manuscript. All authors read and approved the final manuscript.

## CONFLICT OF INTEREST STATEMENT

The authors have no conflicts of interest to report.

## Data Availability

Data sharing is not applicable to this article as no datasets were generated or analyzed during the current study.
